# Enhancement of Precise Gene Editing by the Association of Cas9 With Homologous Recombination Factors

**DOI:** 10.3389/fgene.2019.00365

**Published:** 2019-04-30

**Authors:** Ngoc-Tung Tran, Sanum Bashir, Xun Li, Jana Rossius, Van Trung Chu, Klaus Rajewsky, Ralf Kühn

**Affiliations:** ^1^Max-Delbrück-Centrum für Molekulare Medizin, Berlin, Germany; ^2^Berlin Institute of Health, Berlin, Germany

**Keywords:** Cas9, gene editing, homologous recombination, CtIP, CRISPR

## Abstract

The CRISPR-Cas9 system is used for genome editing in mammalian cells by introducing double-strand breaks (DSBs) which are predominantly repaired via non-homologous end joining (NHEJ) or to lesser extent by homology-directed repair (HDR). To enhance HDR for improving the introduction of precise genetic modifications, we tested fusion proteins of Cas9 nuclease with HDR effectors to enforce their localization at DSBs. Using a traffic-light DSB repair reporter (TLR) system for the quantitative detection of HDR and NHEJ events in human HEK cells we found that Cas9 fusions with CtIP, Rad52, and Mre11, but not Rad51C promote HDR up to twofold in human cells and significantly reduce NHEJ events. We further compared, as an alternative to the direct fusion with Cas9, two components configurations that associate CtIP fusion proteins with a Cas9-SunTag fusion or with guide RNA that includes MS2 binding loops. We found that the Cas9-CtIP fusion and the MS2-CtIP system, but not the SunTag approach increase the ratio of HDR/NHEJ 4.5–6-fold. Optimal results are obtained by the combined use of Cas9-CtIP and MS2-CtIP, shifting the HDR/NHEJ ratio by a factor of 14.9. Thus, our findings provide a simple and effective tool to promote precise gene modifications in mammalian cells.

## Introduction

**FIGURE 1:**
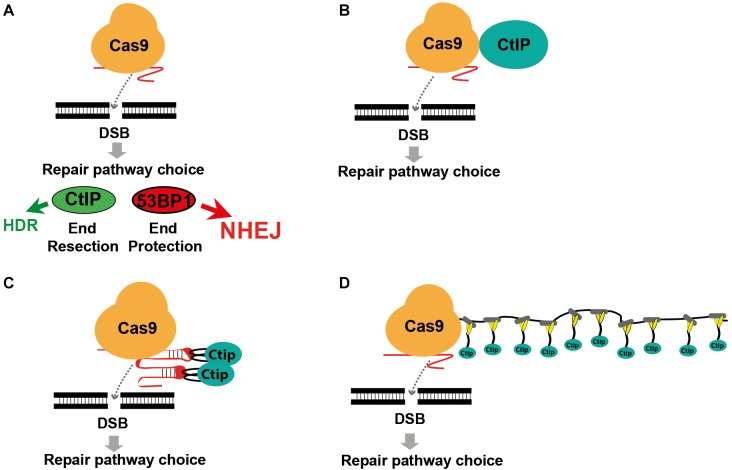
Modifying DSB repair pathway choice by association of Cas9 with CtIP. **(A)** Cas9 induced double-strand breaks are either repaired by HDR or the prevailing NHEJ pathway. DSB repair pathway choice is largely determined by the competition between 53BP1 and BRCA1, triggering either end protection or the CtIP induced resection of DSB ends, followed by the engagement of the NHEJ or HDR pathway. **(B)** Use of a Cas9-CtIP fusion protein for shifting DSB repair choice toward HDR. **(C)** Association of Cas9 and CtIP via sgRNA loops that include MS2 phage aptamer sequences bound by fusion proteins of CtIP and the MS2 coat protein. **(D)** Association of a Cas9-SunTag fusion protein with multiple copies of a SunLigand-CtIP fusion protein.

The RNA guided Cas9 nuclease is used to create targeted double-strand breaks (DSBs) in the genome of mammalian cells and represents a versatile tool for genome editing (Barrangou and Doudna, 2016; Komor et al., 2016). CRISPR-Cas9 mediated DSBs are repaired by either the non-homologous end joining (NHEJ) repair pathway that leads to randomly sized small deletions or insertions (Indels), or by homology-directed repair (HDR) enabling precise sequence modifications that are copied from a repair template. Since HDR requires the presence of a repair template and is restricted to the S and G2 phases of the cell cycle it occurs less frequently than NHEJ. This presents a barrier to applications that rely on precise sequence modifications, such as the correction of mutations in somatic gene therapy or the modeling of disease mutations. To reinforce precise gene editing at Cas9 induced DSBs, tools for shifting the repair pathway choice in favor of HDR must be developed. DSB repair pathway choice is largely determined by the competition between the 53BP1 and BRCA1 regulator proteins, triggering either the protection or resection of DSB ends and the subsequent engagement of the NHEJ or HDR pathway, respectively (Figure 1A; Daley and Sung, 2014; Gupta et al., 2014; Zimmermann and de Lange, 2014). 53BP1 is recruited to DSBs by recognition of the Ubiquitin mark at Lysine 15 of histone H2A (Fradet-Turcotte et al., 2013) in chromatin flanking the break sites. 53BP1 blocks CtIP-based end resection (Bunting et al., 2010) and recruits Rif1 and the Shieldin complex (Mirman et al., 2018), which further block end resection and inhibit BRCA1 accumulation (Escribano-Díaz et al., 2013; Zimmermann et al., 2013). In contrast, the HDR pathway requires the exclusion of 53BP1 and the resection of DSB ends in order to be initiated. During the S/G2 phase, BRCA1 excludes Rif1 from DSB repair foci, and recruits CtIP and the MRE11-Rad50-NBS1 (MRN) complex. This complex initiates a cleavage step which is then further respected at the 5′end by Exo1 (Sartori et al., 2007; Symington and Gautier, 2011; Symington, 2016) extending on each side of the DSB (Zakharyevich et al., 2010). The exposed single-stranded DNA (ssDNA) is protected by binding of RPA1 that is subsequently replaced by Rad51 through the action of BRCA2 and Rad52, forming a nucleofilament competent for homology search and strand invasion (Liu et al., 2011).

Previous approaches to enhance HDR include enrichment of cells in the S/G2 phase (Lin et al., 2014; Yang et al., 2016), restriction of Cas9 activity to the S/G2 phase (Gutschner et al., 2016; Howden et al., 2016), inhibition of the NHEJ key molecules DNA ligase IV (Chu et al., 2015; Maruyama et al., 2015) or 53BP1 (Paulsen et al., 2017; Canny et al., 2018) and the fusion of Cas9 with CtIP (Figure 1B; Charpentier et al., 2018). Here we extended the Cas9 fusion approach to the HDR key proteins Mre11, CtIP, RPA1, Rad51C, and Rad52. Using CtIP, we compared the direct fusion with Cas9 to alternative molecular configurations that associate CtIP with guide RNA that includes MS2 binding loops (Figure 1C) or with a Cas9-SunTag fusion protein (Figure 1D), previously developed for binding of transcriptional activators to dCas9 (Tanenbaum et al., 2014; Konermann et al., 2015). In addition, we compared wildtype CtIP, that is activated in the S/G2 cell cycle phases through phosphorylation at the Threonine residue 847, with the phosphomimetic T847E mutant that is also active in the G1 phase (Huertas and Jackson, 2009). Using a traffic-light DSB repair reporter (TLR) system for the quantitative detection of HDR and NHEJ events in human HEK cells we found that both the fusion of CtIP with Cas9 and the MS2, but not the SunTag system strongly shift the balance of DSB repair pathway choice toward HDR. Best results were obtained by the combined use of both systems, increasing the HDR/NHEJ ratio 14.9-fold.

## Results

### DSB Repair Modification by Cas9 Fusion Proteins

**FIGURE 2:**
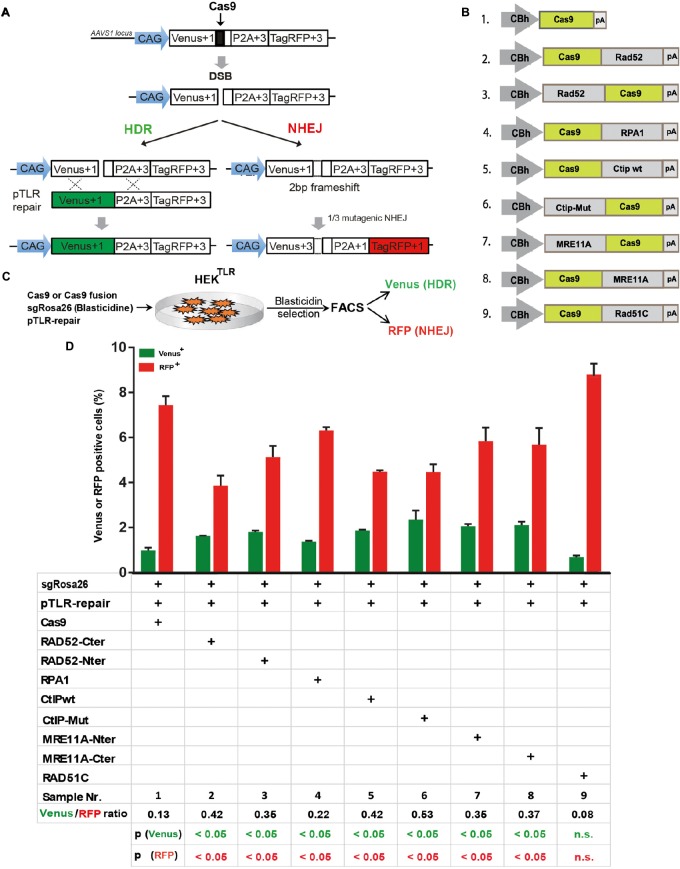
DSB repair modification by Cas9 fusion proteins. **(A)** The traffic light reporter (TLR) construct indicates DSB repair by NHEJ or HDR and was integrated into the AAVS1 locus of HEK cells (HEK^TLR^). Upon induction of a DSB in the defective Venus coding region, RFP is expressed upon NHEJ repair resulting into deletions that shift translation by 2 bp. Venus expression occurs upon HDR with a repair template vector that includes the intact Venus coding region (pTLR-repair). **(B)** Vectors for the expression of Cas9 or fusion proteins between the N- or C-terminal end of Cas9 and Rad52, RPA1, CtIP wildtype (wt), or the T847E mutant (mut), Mre11A or Rad51C, driven by the CBh promoter. pA – polyadenylation signal. **(C)** For DSB repair assays HEK^TLR^ reporter cells were cotransfected with expression vectors for Cas9 or Cas9 fusion proteins, sgRosa26, Blasticidine and pTLR-repair. After 4 days of Blasticidine selection the samples were analyzed by FACS for the presence of Venus and RFP positive cells. **(D)** Bar graph representation of Venus and RFP positive cells, indicating HDR or NHEJ repair of the TLR reporter, upon transfection with an expression vector for Cas9, or Cas9 in C-terminal (Cter) or N-terminal (Nter) fusion with Rad52, RPA1, CtIP wildtype (wt), CtIP mutant T847E (mut), MRE11A, or Rad51C. Bars show mean values of three independent samples with standard deviation. These values were used to calculate the ratio of Venus^+^ to RFP^+^ cells and *p*-values (*T-*test) to determine the significance in levels of Venus^+^ or RFP^+^ cells between samples 1 and 2–9. n.s., not significant, *p* > 0.05.

To quantitatively determine CRISPR/Cas9-induced DSB repair by HDR or NHEJ, we used a traffic light reporter (TLR) construct, integrated into the AAVS1 locus of human HEK293 cells (HEK^TLR^) as previously described (Chu et al., 2015). Briefly, the reporter cassette includes a CAG promoter for expression of a non-functional coding region for Yellow fluorescent (Venus) protein, disrupted by the replacement of codons 117–152 with a 23 bp gRNA target sequence from the mouse *Rosa26* locus (sgRosa26), followed by a P2A peptide and the coding region for a red fluorescent (TagRFP) protein in a reading frame shifted by 2 bp (Figure 2A). If an intact Venus coding sequence is provided as a template and the repair of DSBs occurs via the HDR pathway the reporter cells are detected by the expression of Venus. CRISPR/Cas9-induced DSBs in the target region that are repaired via NHEJ and acquire Indels resulting into the shift of translation into the frame (+2) of P2A-RFP are detectable by the expression of RFP in reporter cells. Analysis by the inDelphi tool (Shen et al., 2018) for the repair of the *Rosa26* target site in HEK293 cells predicts a frequency of 16% of products in the +2 frame. Therefore, Venus positive reporter cells represent all HDR events but the number of RFP positive cells in a sample indicates only a fraction of NHEJ repair events. For the assessment of HDR modifiers we constructed N- or C-terminal fusions of Cas9 with the coding regions of human MRE11A, CtIP (wildtype or the phosphomimetic T847E mutant), RPA1, Rad51C, or Rad52 separated by a flexible linker of 16 residues (Figure 2B). To monitor the effects of Cas9 fusions on DSBs repair pathways, we co-transfected HEK^TLR^ cells with plasmids expressing either Cas9 or Cas9 fusions, a vector for expression of sgRosa26 and a Blasticidine resistance gene together with the donor plasmid (pTLR-repair) for repair of the defective Venus reporter gene (Figure 2C). The transfected cells were selected with Blasticidine for the enrichment of transfected cells and the frequency of Venus^+^ and RFP^+^ cells was analyzed 4 days later by flow cytometry (Supplementary Figure 1) in 4 independent samples. The results were used to calculate mean values and standard deviation. The ratio of Venus^+^ versus RFP^+^ cells is used as a relative index for DSB repair of the reporter by HDR or by NHEJ events resulting into the +2-reading frame. As shown in Figure 2D, upon expression of Cas9 we observed 0.95% of Venus^+^ and 7.55% of RFP^+^ cells in the ratio of 0.13 (sample 1). The expression of a N- or C-terminal fusion protein of Cas9 with Rad52 both lead to the increase of Venus^+^ and the decrease of RFP^+^ cells, shifting the Venus/RFP ratio to values of 0.35 and 0.42, respectively. The expression of a N- or C-terminal fusion protein of Cas9 with MRE11A lead to a higher increase of Venus^+^ cells but a lower decrease of RFP^+^ cells (samples 7 and 8), exhibiting Venus/RFP ratios of 0.35 and 0.37, respectively. The expression of a C-terminal fusion of Cas9 with CtIP or its N-terminal fusion with the CtIP (T847E) mutant (samples 5 and 6) increased the level of Venus^+^ cells up to 2.6%, showing Venus/RFP ratios of 0.42 and 0.53, respectively. In contrast to Rad52, MRE11A, and CtIP, the Cas9-RPA1 fusion protein (sample 4) lead to a smaller shift of the Venus/RFP ratio (0.22). As compared to the control (Cas9, sample 1) the increase of Venus^+^ cells and the decrease of RFP^+^ cells in samples 2–8 was significantly different (*p* < 0.05). Only the expression of the Cas9-Rad51C fusion (sample 9) resulted into levels of Venus^+^ cells and RFP^+^ cells that were not significantly different from the control (sample 1). These results show that the use of Cas9 fusions with multiple proteins of the HDR pathway, specifically CtIP, MRE11A and Rad52, can be used to stimulate DSB repair by HDR up to 2.7-fold.

### Cas9 Fusion Proteins in 53BP1 Knockout HEK^TLR^ Cells

**FIGURE 3:**
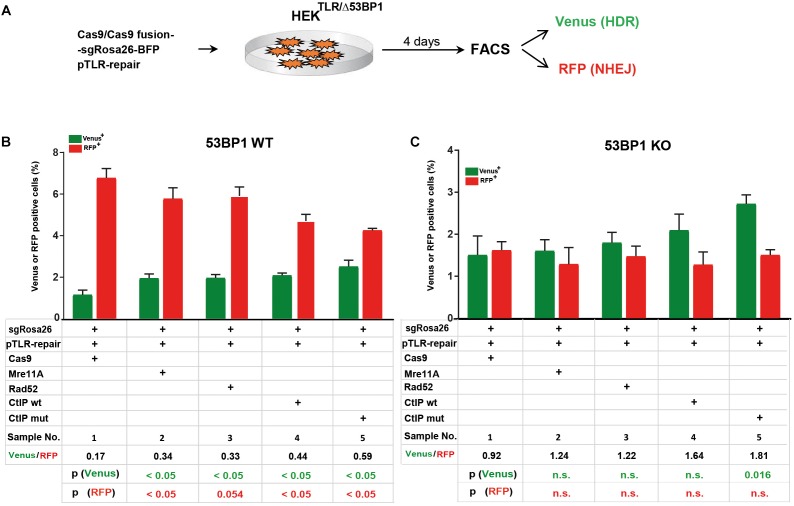
Cas9 fusion proteins in 53BP1 knockout HEK^TLR^ cells. **(A)** For DSB repair assays in HEK^TLR^ and HEK^TLR^ cells harboring 53BP1 knockout alleles (HEK^TLR/Δ53BP1^), reporter cells were cotransfected in duplicate with expression vectors for Cas9 or Cas9 fusion proteins, sgRosa26, BFP and pTLR-repair. After 4 days the samples were analyzed by flow cytometry (FACS) for the presence of Venus and RFP positive cells indicating repair of the TLR construct by HDR or NHEJ. **(B)** Bar graph representation of Venus and RFP positive cells, indicating HDR or NHEJ repair of the reporter in HEK^TLR^ wildtype (53BP1-WT) cells or **(C)** HEK^TLR/Δ53BP1^ (53BP1-KO) cells, upon transfection with an expression vector for Cas9, or Cas9 in C-terminal fusion with MRE11A, Rad52, CtIP wildtype (wt), or the CtIP mutant T847E (mut). Bars show mean values of four (samples 1–3) or three (samples 4, 5) independent samples with standard deviation. These values were used to calculate the ratio of Venus^+^ to RFP^+^ cells and *p*-values (*T*-test) to determine the significance in levels of Venus^+^ or RFP^+^ cells between samples 1 and 2–5. n.s., not significant, *p* > 0.05.

The 53BP1 key regulator of NHEJ as well as its interaction partners Rif1 and the shieldin complex counteract DSB end resection. In order to elucidate the role of 53BP1 in the TLR assay with Cas9 fusion proteins we generated reporter cells harboring frameshift knockout alleles of the 53BP1 gene (HEK^TLR/Δ53BP1^) using CRISPR-Cas9 (Supplementary Figure 2A). HEK^TLR^ and HEK^TLR/Δ53BP1^ reporter cells were transfected with expression vectors for Cas9 or Cas9 fusions, the sgRosa26 and a BFP reporter together with pTLR-repair (Figure 3A). The BFP^+^ population representing the transfected cells was analyzed 4 days later by flow cytometry (Supplementary Figure 2B) to determine the frequency of Venus^+^ and RFP^+^ cells. The results were used to calculate mean values shown in bar plots with standard deviation. As shown in Figure 3B, in HEK^TLR^ cells with wildtype alleles of 53BP1 the expression of Cas9-MRE11A, -Rad52, or -CtIP lead to a statistically significant (*p* < 0.05) increase of Venus^+^ cells and a decrease of RFP^+^ cells, as observed previously (Figure 2D). In contrast, the transfection of Cas9 into 53BP1 deficient HEK^TLR/Δ53BP1^ cells lead to twofold higher levels of Venus^+^ cells and a more than threefold reduction of RFP^+^ cells to 1.7% (Figure 3C, sample 1), as compared to 6.73% RFP^+^ in HEK^TLR^ cells (Figure 3B, sample 1). As compared to HEK^TLR^ cells the transfection of HEK^TLR/Δ53BP1^ cells with Cas9-Mre11, -Rad52, and -CtIP wildtype fusion proteins did not significantly increase the levels of Venus^+^ cells (samples 2–4), whereas the levels of RFP^+^ cells fall below 2% and was not significantly different from the control (sample 1). Only the expression of Cas9-CtIP^T847E^ fusion protein lead to a significant increase of Venus^+^ cells. These results suggest that in wildtype HEK^TLR^ cells Cas9 fusion proteins with MRE11A, Rad52, or CtIP are efficiently counteracting the inhibitory action of 53BP1 on DSB end resection, leading to increased HDR repair. Nevertheless, Cas9 fusion proteins interfere only weakly with the NHEJ promoting activity of 53BP1 since the numbers of RFP^+^ cells in wildtype HEK cells were only moderately suppressed, but strongly reduced in 53BP1 knockout cells. In conclusion, we found that Cas9 fusions with MRE11A, Rad52, and CtIP are useful tools for increasing HDR mediated gene editing but only weak suppressors of NHEJ. Thus, experimental conditions for the concurrent increase of HDR together with NHEJ suppression would require an additional, active inhibition of 53BP1.

### Targeting of the Beta-2 Microglobulin Gene in HEK Cells

**FIGURE 4:**
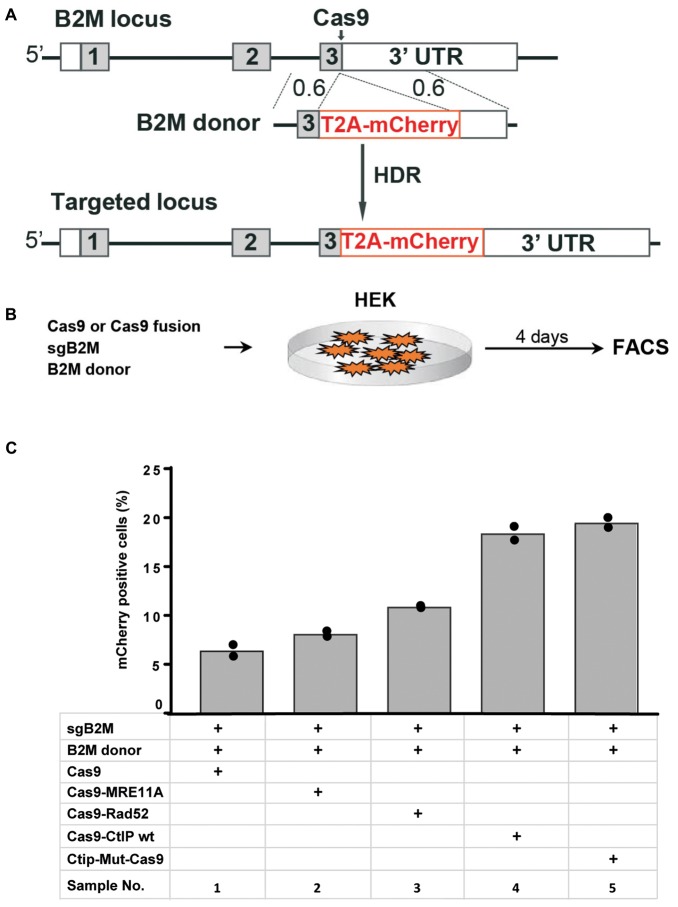
Targeting of the beta-2 microglobulin gene **(A)** The beta-2 microglobulin locus (B2M) was targeted in HEK cells by HDR mediated insertion of a T2A-mCherry coding region placed upstream of the B2M Stop codon and 3′untranslated region (3′-UTR) using a donor vector with 600 bp homology regions (0.6). **(B)** Four days after the transfection of HEK cells with expression vectors for Cas9 or Cas9 fusion protein, sgRNA against the B2M target region and the donor vector, the frequency of Cherry positive cells was determined by flow cytometry (FACS). **(C)** Bar graph representation of Cherry positive cells, indicating HDR at the B2M locus upon transfection with an expression vector for Cas9, or Cas9 in C-terminal fusion with MRE11A, Rad52, CtIP wildtype (WT), or the CtIP mutant T847E (mut). Bars show mean values of four independent samples with standard deviation. These values were used to calculate *p*-values (*T*-test) to determine the significance in levels of cherry^+^ cells between samples 1 and 2–5.

In addition to the TLR reporter construct we assessed the effect of Cas9 fusion proteins on targeting a red fluorescent mCherry reporter gene into the last exon of the beta-2 microglobulin (B2M) gene of HEK cells. For targeting of the B2M locus we used a sgRNA against B2M (sgB2M) and a repair template vector for HDR (B2M-donor) that leads to the insertion of a 2A peptide and the mCherry coding region upstream of the B2M Stop codon (Figure 4A). HEK cells were transfected with expression vectors for Cas9 or Cas9 fusions, sgB2M and the B2M donor vector (Figure 4B). The transfected cell population was analyzed 4 days later by flow cytometry for the presence of Cherry^+^ cells (Supplementary Figure 3). The results were used to calculate mean values of 4 independent samples with standard deviation. As shown in Figure 4C, the transfection with Cas9, sgB2M, and B2M donor resulted into 6.4% Cherry^+^ cells. The use of expression vectors for Cas9-MRE11A or Cas9-Rad52 fusion protein moderately, but significantly (*p* < 0.05) increased the frequency of Cherry^+^ cells to 8.1 or 10.9%, whereas the expression of Cas9-CtIP or Cas9-CtIP(T847E) fusion protein more than doubled the number of Cherry^+^ cells to 18.4 or 19.5%, respectively (Figure 4C). These results show that in particular the Cas9-CtIP fusion proteins are able to support HDR at the endogenous B2M target locus in HEK cells.

### Association of CtIP and Cas9 via the MS2 and SunTag Systems

**FIGURE 5:**
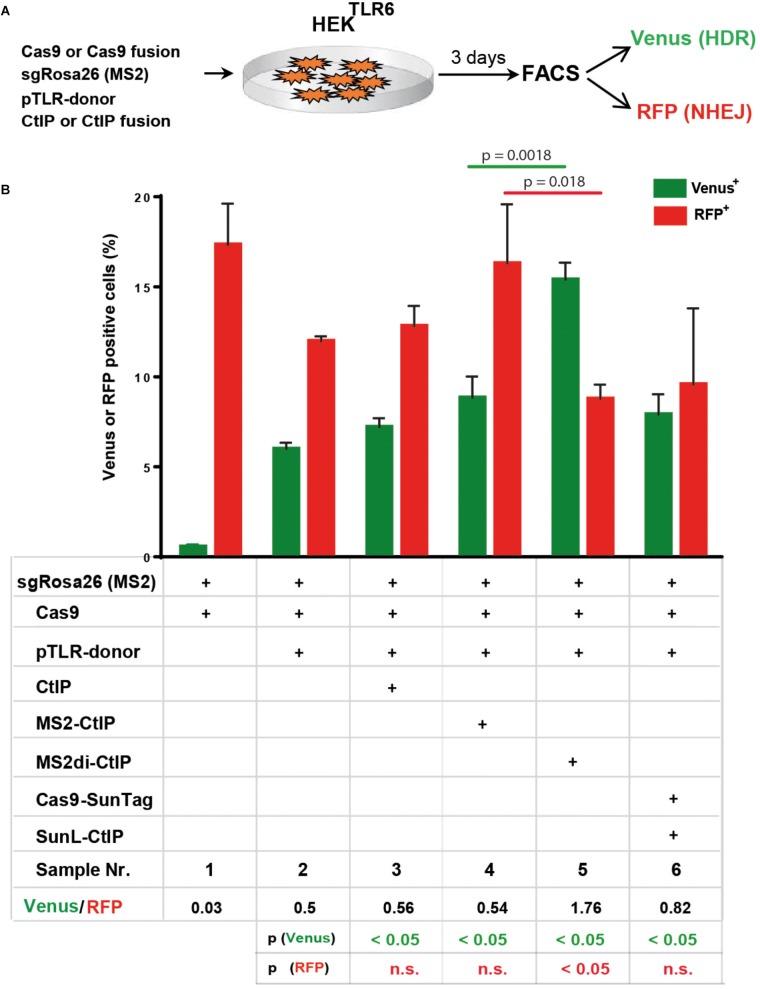
DSB repair modification by MS2-CtIP and SunL-CtIP fusion proteins in HEK^TLR6^ reporter cells. **(A)** Expression vectors for Cas9 or Cas9-SunTag, sgRosa26(MS2), pTLR-donor and CtIP, MS2-CtIP, MS2^di^-CtIP, or SunL-CtIP were cotransfected into HEK^TLR6^ cells and the frequency of Venus^+^ cells (green columns) and RFP^+^ cells (red columns), indicating DSB repair by HDR or NHEJ, was determined by flow cytometry (FACS) 72 h after transfection. **(B)** Transfection results are shown as bar graphs representing mean values with standard deviation of triplicate samples. Plasmids selected (+) for individual transfection samples are indicated in the table below. Mean values were used to calculate the ratio of Venus^+^ to RFP^+^ cells and *p*-values (*T*-test) to determine the significance in levels of Venus^+^ or RFP^+^ cells between samples 2 and 3–6 (table bottom) and to compare samples 4 and 5 (top horizontal lines). n.s., not significant, *p* > 0.05. Two independent replicates of the assay were performed that confirmed the results.

Besides Cas9-CtIP fusion proteins we further assessed the MS2 and the SunTag system as alternative molecular configurations for the association of CtIP to Cas9 induced DSBs (Figure 1C,D). In a first approach we fused CtIP with a monomer of the MS2 bacteriophage coat protein (MS2-CtIP) which binds as dimer to a MS2 derived 34 nt RNA aptamer sequence motif that can be included in the tetraloop and stem loop of sgRNAs, as described (Konermann et al., 2015; Supplementary Figures 4A,B). Since CtIP must associate via its N-terminus into a tetramer to become functional, the binding of two closely neighbored MS2-CtIP molecules at each MS2-sgRNA loop (Supplementary Figure 4B). may be inefficient or interfere with its tetramerization. Therefore, we also constructed a fusion of CtIP with a single chain MS2 dimer (MS2^di^-CtIP), that binds to the MS2 aptamer RNA as a monomer (Peabody and Lim, 1996; Supplementary Figure 4C). In a second approach Cas9 was fused with 10 repeats of a GCN4 derived peptide motif (Cas9-SunTag) (Tanenbaum et al., 2014) that is recognized by a high affinity single-chain antibody, designated here as SunLigand (SunL) (Supplementary Figure 4D). For the association of CtIP with Cas9-SunTag we constructed an expression vector for a SunL-CtIP(T847E) fusion protein. For these assays we used HEK reporter cells harboring in the AAVS1 locus a modified TLR reporter construct (HEK^TLR6^) that includes a Venus coding region disrupted by the replacement of codons 95–97 with the same *Rosa26* derived target sequence used in the TLR reporter. Since HEK^TLR6^ cells are homozygous for the reporter construct a small population of double positive cells appears upon DSB induction, undergoing HDR repair on one reporter allele and a mutagenic NHEJ event on the other. For DSB repair assays the number of single positive Venus and RFP positive cells was determined, excluding double positive cells. For repair of the TLR6 reporter we used a template vector (pTLR-donor) that includes the Venus coding sequence, excluding the start codon to prevent background expression. HEK^TLR6^ cells were cotransfected with plasmids for expression of Cas9, Cas9-CtIP or Cas9-SunTag driven by the CAG promoter together with pTLR-donor and the *Rosa26* specific sgRNA including two MS2 aptamer sequences [sgRosa26(MS2]. Three days after transfection the frequency of Venus^+^ and RFP^+^ cells from triplicate samples was determined by flow cytometry and the mean values and standard deviations were calculated (Figure 5A). The ratio of Venus^+^/RFP^+^ cells is used as an index for DSB repair choice by HDR or NHEJ. Of note, this relative value represents the ratio of all reporter HDR events to only a fraction of NHEJ mediated Indels that reconstitute the RFP reading frame. As shown in Figure 5B, the transfection of Cas9, sgRosa26(MS2) and pTLR-donor resulted into 5.6% Venus and 11.4% RFP cells (sample 2; Venus/RFP ratio: 0.5), whereas a control without pTLR-donor (sample 1) showed a Venus background of 0.4%. The expression of Cas9 and free CtIP (sample 3) yielded 6.8% Venus and 12.2% RFP cells, showing a similar ratio (0.56) as the sample with Cas9 alone. The expression of MS2-CtIP(T847E) lead to a moderate, but statistically significant increase of Venus^+^ cells with a ratio of 0.54 (sample 4). In contrast to MS2-CtIP(T847E), the expression of MS2^di^-CtIP(T847E) strongly increased (*p* = 0.0018) the Venus^+^ cells to 14.6% and reduced the RFP^+^ cells (*p* = 0.018) to 8.3%, resulting into a Venus/RFP ratio of 1.76 (sample 5). In contrast to MS2^di^-CtIP, the expression of Cas9-SunTag and SunL-CtIP did not increase the Venus^+^ cells (sample 6, ratio: 0.82), as compared to the sample with free CtIP. These results show that in fusion with CtIP only the MS2^di^, but not the MS2 or the SunTag configuration, leads to a strong shift of the HDR/NHEJ balance upon DSB repair of the reporter. Since the MS2 and SunTag systems assemble two or more CtIP molecules in close proximity these configurations may interfere with CtIP oligomerization and the stimulation of end resection.

### Comparison of Cas9-CtIP, MS2^di^-CtIP, and SunL-CtIP Fusion Proteins

**FIGURE 6:**
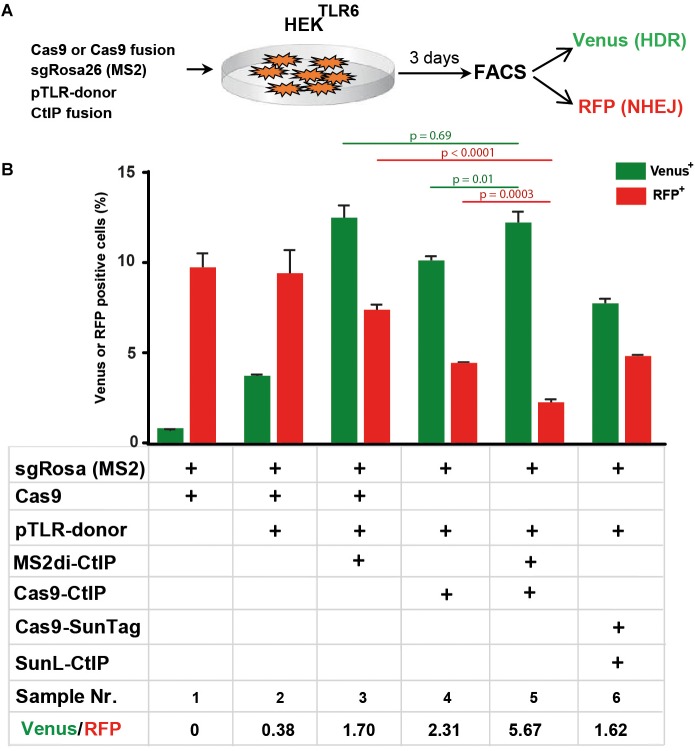
Comparison of Cas9-CtIP, MS2^di^-CtIP, and SunL-CtIP fusion proteins. **(A)** Expression vectors for Cas9, Cas9-CtIP, or Cas9-SunTag, sgRosa26(MS2), pTLR-donor, and MS2^di^-CtIP or SunL-CtIP were cotransfected into HEK^TLR6^ cells and the frequency of Venus^+^ cells (green columns) and RFP^+^ cells (red columns), indicating DSB repair by HDR or NHEJ, was determined by FACS analysis 72 h after transfection. **(B)** Transfection results are shown as bar graphs representing mean values ± standard deviation of triplicate samples. Plasmids selected (+) for individual transfections are indicated as in the table below. Mean values were used to calculate the ratio of Venus^+^ to RFP^+^ cells and *p*-values (*T-*test) to determine the significance in levels of Venus^+^ or RFP^+^ cells between samples 5 and 3 or 4 (top horizontal lines). Two independent replicates of the assay were performed that confirmed the results.

Next, we compared the performance of the Cas9-CtIP fusion protein with the MS2^di^-CtIP and SunL-CtIP systems in HEK^TLR6^ cells to determine which approach is most effective for HDR stimulation. For a side-by-side comparison we transferred the Cas9-CtIP coding region into the same vector used for Cas9 expression in MS2^di^-CtIP assays that includes a CAG promoter region and the sgRosa26(MS2) expression cassette (Figure 6A). As shown in Figure 6B, the transfection of Cas9, sgRosa26(MS2), and pTLR-donor resulted into 3.6% Venus and 9.2% RFP cells (sample 2; ratio: 0.38), whereas a control without pTLR-donor (sample 1) showed a Venus background of 0.7%. The expression of MS2^di^-CtIP (sample 3) yielded 12.4% Venus and 7.2% RFP cells, showing a Venus/RFP ratio of 1.7. The expression of the Cas-CtIP fusion lead to a further increase of the Venus/RFP ratio to 2.31 although the absolute levels of Venus and RFP cells were decreased (sample 4). Interestingly, the combined expression of Cas-CtIP and MS2^di^-CtIP lead to a further decrease of RFP^+^ cells to 2.1% (sample 5), resulting into an increased ratio of 5.67. The expression of Cas9-SunTag and SunL-CtIP lead to a Venus/RFP ratio of 1.62, but lower levels of Venus^+^ and RFP^+^ cells as compared to the sample with MS2^di^-CtIP. These results show that both Cas9-CtIP and MS2^di^-CtIP are shifting the ratio of HDR/NHEJ repair of the reporter construct. Best results were obtained when both systems were combined, leading to a significant decrease of RFP^+^ cells (*p* < 0.05) as compared to the use of Cas9-CtIP or MS2^di^-CtIP alone, shifting the HDR/NHEJ balance of DSB repair at the reporter by a factor of 14.9.

## Discussion

For the improvement of HDR and precise gene editing at Cas9 induced DSBs we compared three approaches for associating HDR effector proteins with the nuclease or guide RNA. Firstly, we tested the ability of Cas9/HDR effector fusion proteins and found that CtIP but also Rad52 and MRE11A are effective for HDR stimulation, counteracting the inhibition of end resection by 53BP1 without strong suppression of NHEJ. Using CtIP as paradigm we compared the Cas9-CtIP fusion to methods based on anchoring of SunL-CtIP to a Cas9-SunTag protein or MS2-CtIP fusions to gRNA that includes MS2 recognition sequences. Our results show that the performance of the single chain MS2^di^-CtIP fusion was comparable to the Cas9-CtIP direct fusion and that the combination of both proteins had an additive effect on NHEJ suppression, shifting the HDR/NHEJ repair of the reporter construct by a factor of 14.9. Our findings provide simple and effective tools to promote precise gene modifications in mammalian cells.

Previous studies based on HDR stimulation rarely explored the association of HDR effectors with Cas9/gRNA complexes, except for the work of Charpentier et al. (2018) that described the N-terminal fusion of CtIP with Cas9. In this study it was found that CtIP-Cas9 increased HDR at the AAVS1 locus in human cells twofold without reducing Indel formation and that the N-terminal oligomerization domain of CtIP is sufficient for HDR stimulation. Our results extend the analysis of Cas9-fusion proteins to the HDR effectors MRE11A and Rad52 and confirm the previous finding that the N-terminal but also the C-terminal fusion of CtIP to Cas9 stimulates HDR. In addition, we validated the MS2 aptamer system as a useful tool for HDR stimulation by the association of CtIP with sgRNA loops. In fusion with CtIP the MS2^di^ single chain configuration was more effective than MS2 monomers that require dimer formation for binding to the MS2 RNA aptamer. Whereas the MS2 tagged sgRNA provides two binding sites for MS2 fusion proteins, the SunTag system has previously shown utility for signal amplification by binding of up to 24 SunL-GFP molecules to a Cas9-SunTag fusion protein (Tanenbaum et al., 2014). In fusion with CtIP and Cas9-SunTag providing 10 SunL binding sites, we found the SunTag approach was less effective for HDR stimulation than the MS2 system, which might be caused by an interference with CtIP oligomerization in the presence of multiple localized molecules. A similar effect may account for the improved performance of the MS2^di^ single chain dimer as compared to the use of MS2-CtIP monomers. Nevertheless, it remains to be investigated whether MS2 monomers or the SunTag system provide valid options for the use of other HDR effectors which do not form oligomers.

Remarkably, we observed that the combined use of Cas9-CtIP and MS2^di^-CtIP had a cumulative effect on NHEJ suppression but not on HDR stimulation, pointing to the enhanced counteraction against the initiation of NHEJ through 53BP1 by localization of three instead of two or a single CtIP molecule at the Cas9/sgRNA complex. This observation may allow further improvements of the toolbox for manipulation of the DSB repair pathway choice by the assembly of two cooperative HDR effector proteins at the DSB site, such as CtIP together with Rad52, MRE11A or other factors. Furthermore, it may be possible to combine the N-terminal CtIP HDR enhancer domain with one or more active domains from other effectors into multifunctional fusions that are able to bypass rate limiting steps of HDR initiation or HDR processing. Besides the DSB repair pathway choice, the availability of donor templates at DSBs represents a limiting factor for the completion of HDR. It has been previously shown that HDR can be enhanced by the co-localization of donor templates and Cas9 via covalent linkage using the SNAP Tag in fusion with Cas9 that couples to oligodeoxynucleotides modified with Benzylguanin (Savic et al., 2018). Therefore, it will be interesting to explore whether the colocalization of donor templates and of HDR effectors at DSBs by using Cas9 fusion proteins together with the MS2 aptamer system has a synergistic effect on HDR enhancement.

In summary, here we employed a TLR reporter system for the assessment of DSB repair by HDR and NHEJ in HEK cells that we had used earlier to validate DNA Ligase IV as a target for NHEJ suppression (Chu et al., 2015). Although we expect that HDR enhancement at the TLR reporter system is predictive for other genomic targets as previously shown and we indeed confirmed the effect of Cas9-CtIP at the B2M gene, the Cas9 fusion and the MS2 aptamer approach will require further validation using additional target genes and other cell types. For reporting NHEJ activity, the present configuration of the TLR construct has a restriction since it reports only a fraction of NHEJ repair events that restore the RFP reading frame. To increase the utility of TLR reporter constructs in future, modified constructs are required enabling to report NHEJ repair products that occur in all three reading frames. Furthermore, we did not discriminate between DSB repair choice in the G1 and S/G2 cell cycle phases. We anticipate that HDR enhancement occurs primarily in the S/G2 phases in which the HDR pathway usually operates. It has been shown that HDR can be at least partially reactivated in G1 by the combined suppression of 53BP1 and the expression of a degradation resistant Palb2 mutant together with the phosphomimetic CtIP (T847E) mutant (Orthwein et al., 2015). Thus, it will be interesting to determine the effect of Cas9 and MS2 fusions with HDR effectors specifically on DSB repair in the G1 phase and whether the current protocol or further modifications will enable HDR mediated DSB repair in the G1 phase or even in resting cells.

In the current format of using plasmid-based expression vectors we expect that the CtIP co-localization approach can readily support applications of precise gene editing in human cell lines such as modeling or correcting disease-causing mutations by cotransfection with Cas9/sgRNA vectors. In combination with recombinant proteins its applications may be extended in future to primary human cells such as hematopoietic stem cells, muscle satellite or other stem cells exhibiting sufficient basal levels of HDR to assess its utility for the precise correction of mutations required for somatic gene therapy. Since DSB repair mechanism are conserved in evolution, we expect that the co-localization approach using CtIP or other HDR effectors can also be applied for HDR enhancement in other species.

## Materials and Methods

### Plasmid Constructions

In the first set of experiments (Figure 2–4) we used Cas9 fusion vectors that were constructed based on the pX330 vector backbone (Addgene ID 42230), including a CBh promoter for protein expression. First, an oligonucleotide encoding the 16-mer flexible linker (SGSETPGTSESATPES) and multiple cloning sites (PacI and SalI) was in-frame cloned into either the N- or C-terminal end of Cas9. Then, cDNA of DNA repair factors (RAD51, RAD52, CtIP, CtIP_mutant, MRE11A, NBN, RPA1, and RAD50) were cloned between the PacI and SalI sites by standard PCR cloning methods. All primers for cloning are listed in Table 1. sgRNAs against 53BP1 was purchased as separated oligos, phosphorylated, annealed, and cloned into pX330 by standard cloning technique. pTLR-repair (Addgene 64322) was used as repair template vector for DSB repair assays in HEK^TLR^ reporter cells (Chu et al., 2015). In the second set of experiments (Figure 5, 6) we used pU6Rosa-CAG-Cas9 for expression of Rosa26 sgRNA and of Cas9 from the CAG promoter, constructed by ligation of oligonucleotides sgRosa-A/-B into the BbsI sites downstream of a human U6 promoter into plasmid pU6(BbsI). The U6-sgRosa cassette was recovered as AscI fragment and inserted into pCAG-Cas9-bpA-EF1-BFP, upstream of the CAG promoter driving Cas9 expression, followed by a BFP coding region under control of the human EF1α promoter. For cloning of pCAG-Cas9-CtIP a SphI fragment was isolated from pX330-CBh-Cas9-CtIP and ligated between the SphI sites of pCAG-Cas9-bpA-EF1-BFP. Plasmid pTLR-donor was generated by whole plasmid PCR amplification using 5′-phosphorylated primers TLRtv-1 and TLRtv-2 and pTLR-repair as template, followed by of the PCR fragment. The modification of pTLR-repair removes the Start codon of the Venus coding region, eliminating background fluorescence upon transient transfection. Plasmid pU6Rosa-CAG-CtIP(T847E)-EF1-BFP was generated by cloning of a PCR product, amplified with the primers CtIP-for/-rev using as template pCW-GFP-CtIP-T847E (Addgene ID 71111), into the PacI/NotI sites of pU6Rosa-CAG-Pac-Not-EF1BFP. Plasmid pU6Rosa(MS2) was cloned by ligation of oligonucleotides sgRosa-A/-B into the BbsI sites downstream of a human U6 promoter into plasmid sgRNA(MS2) (Addgene ID 64124). The U6Rosa(MS2) cassette was recovered by PCR (primers U6MS2-for/-rev) and cloned in between the AscI sites of pU6Rosa-CAG-CtIP(T847E)-EF1-BFP, replacing the U6Rosa cassette, to derive plasmid pU6Rosa(MS2)-CAG-CtIP(T847E)-EF1-BFP. The latter plasmid was digested with PacI upstream of the CtIP coding region for the in frame insertion of the MS2 coat protein coding region, amplified with primers MS2-for/-rev from plasmid MS2-P65-HSF1_GFP (Addgene ID 61423), to complete the pU6Rosa(MS2)-CAG-MS2-CtIP(T847E)-EF1-BFP vector. Alternatively a 875 bp synthetic gene fragment from pMS2-dimer (Thermo Scientific) encoding a single chain dimer of the MS2 protein (Peabody and Lim, 1996) was cloned into the PacI site of pU6Rosa(MS2)-CAG-CtIP(T847E)-EF1-BFP to obtain the pU6Rosa(MS2)-CAG-MS2^di^-CtIP(T847E)-EF1-BFP vector. The SunLigand single chain antibody coding region was amplified using primers SunLigand-for/-rev from pHRdSV40-scFv-GCN4-sfGFP-VP64-GB1-NLS (Addgene ID 60904) and cloned into the PacI and AfeI site of pMS2-dimer to derive plasmid pSunLigand. Next, the SunLigand coding region was recovered by PCR (primers SunLigand-for, MS2-rev) and cloned into the PacI site of pU6Rosa-CAG-CtIP(T847E)-EF1-BFP in frame with CtIP to derive pU6Rosa-CAG-SunL-CtIP-EF1-BFP. Plasmid pCAG-Cas9-SunTag-bpA was cloned by ligation of a 1483 bp PCR product, amplified from pHRdSV40-dCas9-10xGCN4_v4-P2A-BFP (Addgene ID 60903) using primers Bsm-up and SunTag, into the BsmI/MluI sites of pCAG-Cas9v3a-bpA. pAAVS1-TLR6 was constructed by cloning of a fusion PCR product from a PCR fragment (using primers TLRvenus-1 and TLR6-1) and a PCR fragment (using primers TLR6-2 and TagRFP-2), using pAAVS1-TLR donor (Addgene ID 64215) (Chu et al., 2015) as template, into the backbone of plasmid pCAG-venusTarget+1P2A+3TagRFP (opened with PacI and MluI), resulting into pCAG-TLR6. pCAG-TLR6 was used for isolation of a AscI-AsiSI fragment that was ligated into pAAVS1-TLR donor, resulting into pAAVS1-TLR6, serving as AAVS1 targeting vector with the TLR6 reporter insert.

**Table 1:** Oligodeoxynucleotides.

Name	Sequence (5′–3′)
16mer Linker	AGCGGCAGCGAGACCCCCGGCACCAGCGAGAGCGCCACCCCCGAGAGC
PacI-hCtIP F	ATTTAATTAAAAACATCTCGGGAAGCAGCTGTGGAAGCCC
SalI-hCtIP R	GGC GTCGAC CTATGTCTTCTGCTCCTTGCCTTTTGGAG
PacI-hRAD51 F	AT TTAATTAAA GCAATGCAGATGCAGCTTGAAGCAAATGC
SalI-hRAD51 R	GGC GTCGAC TCAGGAAGACAGGGAGAGTCGTAGATTTTGC
PacI-hMRE11A F	AT TTAATTAAA AGTACTGCAGATGCACTTGATGATGAAA
SalI-hMRE11A R	GGC GTCGAC TTATCTTCTATTTCTTCTTAAAGAACTAGTG
PacI-hNBN F	AT TTAATTAAA CAGAATGGCTTTTCCCGAACTTTGAAGTCGG
SalI-hNBN R	GGC GTCGAC TTATCTTCTCCTTTTTAAATAAGGATTGTATCT
PacI-hRAD50 F	AT TTAATTAAA TCCCGGATCGAAAAGATGAGCATTCTGG
SalI-hRAD50 R	GGC GTCGAC TTAATGAACATTGAATCCCAGGGAGCT
PacI-hRAD52 F	ATTTAATTAAA TCTGGGACTGAGGAAGCAATTCTTGGA
SalI-hRAD52 R	GGC GTCGAC TTAAGATGGATCATATTTCCTTTTCTTCATGTCCTGGC
sgRNA_53BP1_Exon1.1	AGATTCTCAGCCTGAAAGCC
sgRNA_53BP1_Exon1.2	GCCTGAAAGCCAGGTTCTAG
sgRNA_53BP1_Exon2.1	GCTGGAGAAGAACGAGGAGA
sgRNA_53BP1_Exon2.2	GAACGAGGAGACGGTAATAG
T7_h53BP1_F	GAGGAATGGTGACTTGGTAGGCATGATAGG
T7_h53BP1_R	TGACCTGACTGATGGAACCACATG
TLRvenus-1	ATCCTTAATTAAGCCGCCACCATGGTGAGCAAGGGCGAGGAGCTG
TLR6-1	CCATCTTCTAGAAAGACTGGAGTCTGGACGTAGCCTTCGGGCATG
TLR6-2	GACTCCAGTCTTTCTAGAAGATGGATCTTCTTCAAGGACGACGGC
TagRFP-2	ATTTACGCGTGCGATCGCTCAATTAAGTTTGTGCCCCAGTTTG
TLRtv-1	GTGAGCAAGGGCGAGGAGCTG
TLRtv2	GGTGGCGGCTTAATCAAGCTTATC
sgRosa-A	CACCGACTCCAGTCTTTCTAGAAGA
sgRosa-B	AAACTCTTCTAGAAAGACTGGAGTC
i53-for	TCCTTAATTAATAATACGACTCACTATAGGGGCCACCATGTTGATTTTCGTGAAAACCCTTAC
i53-rev	CATTTACGCGTTCAACGAAGTCTCAACAGAGGATG
CtIP-for	GATCCTTAATTAATAATACGACTCACTATAGGGGCCACCATGAACATCTCGGGAAGCAGCTG
CtIP-rev	TCATTTACGCGTCTATGTCTTCTGCTCCTTGCCTTT
U6MS2-for	TGAGGGGCGCGCCCGAGGGCCTATTTCCCATGATTCC
U6MS2-rev	GAATCCGGCGCGCCAAAAAAAGCACCGACTCGGTGCCAC
MS2-for	GATCCTTAATTAAGCCACCATGGCTTCAAACTTTACT
MS2-rev	ATGATCGATCGAGCGGCCGCCACCTTCCTCTT
SunLigand-for	ATTAAGCCACCATGGGCCCCGACATCGTGATG
SunLigand-rev	CCTCCAGCGCTATCGCCAATTGGAGTATTTTGTTG
Bsm-up	CCTGTTCGAGCTGGAAAACGGC
SunTag	ATTTACGCGTTATCATCCCACCTTGCGCTTCTTCTTGGGTCCACCTC
ST_puro_gt_fw	TCCCCTCTTCCGATGTTGAG
ST_puro_gt_rv	GCGGTCCGGATCGACG


### Generation of HEK Reporter Cells

The generation of HEK^TLR^ cells was previously described (Chu et al., 2015). Cells were cultured in DMEM (Gibco) supplemented with 10% FBS (Gibco), 2 mM sodium pyruvate (Gibco), 2 mM L-glutamine (Gibco), and 1× NEAA (Gibco). Cells were maintained in the exponential phase. Transfected cells with Blasticidin-resistant vector were selected with 10 ug/ml of blasticidin for 4 days before analysis. The HEK^TLR6^ line was maintained in Dulbecco’s Modified Eagle’s medium with Glutamax (Gibco) supplied with 10% fetal bovine serum (Gibco) and generated by transfection of pAAVS1-TLR6 together with sgRNA plasmid against AAVS1 and a Cas9 plasmid (750 ng each) using Xtreme-gene transfection reagent (Roche). Antibiotic selection was performed using 0.4 μg/ml Puromycin. Single clones were generated and genotyped using genomic DNA isolated using the Wizard genomic DNA purification kit (Promega #A1125). PCR was performed for knock-in of the TLR6 construct (puro 5′) as well as the AAVS1 WT locus specific as a control. The PCR reaction for TLR Knockin was performed using primers ST_puro_gt_fw and ST_puro_gt_rv. Phusion HF DNA polymerase (NEB # M0530 L) and 200 ng genomic DNA using following conditions; initial denaturation at 98°C for 3 min, followed by 35 cycles of 98°C 30 s, 58°C 30 s and 72°C for 90 s. final extension was done at 72°C for 5 min. AAVS1 WT PCR was performed using primers hAAVS1-For and hAAVS1-Rev by amplifying at 98°C for 5 min, followed by 40 cycles of 98°C 30 s, 60°C 30 s, 72°C for 45 s and final extension at 72°C for 5 min. After confirmation of TLR insertion by genotyping, selected clones were tested for activity of the TLR allele by FACS-based assay and a single clone was chosen for further assays. All cell lines were confirmed for the absence of mycoplasma using the PCR assay of Uphoff and Drexler (2002).

### Targeting of the 53BP1 Gene in HEK^TLR^ Cells

sgRNAs against Exon1 and 2 of the 53BP1 gene were designed using the CrispRGold program^1^ and cloned into the pX330 vector using the oligonucleotides sgRNA_53BP1_exon1.1/-1.2 and _exon2.1/-2.2. Plasmid px330-sgRNA_exon1.1 and -sgRNA_exon2.2 were co-transfected into HEK^TLR^ cells to generate TP53BP1 knockout subclones. Three days post-transfection, targeted HEK^TLR^ cells were sorted as single cells into 96-well plates, cultured and expanded for genotyping by PCR using primers T7_h53BP1 primers listed in Table 1. Deletion of the 53BP1 gene segment between the Cas9 target sites in the first and second exon was confirmed by PCR by sequencing of the PCR product. Clone 7 that showed homozygous deletions (Supplementary Figure 2A) was selected for further experiments.

### DSB Repair Assays

HEK^TLR^ or HEK^TLR/Δ53BP1^ cells were plated into 6-well plates 1 day before transfection at a density of 1 × 10^5^ cells/well. Transfection was carried out using FuGENE HD Reagent (Promega) according to the manufacturer’s introduction. Briefly, DNA was diluted in OptiMEM medium (GIBCO), then FuGENE was added in the mixture with the ratio of 3:1 (FuGENE:DNA). The mixture was incubated at room temperature for 10 min and then dropped slowly into pre-plated cells. Next day, medium was changed to the stop reaction. We used the same molar ratio of plasmids for transfection if multiple plasmids were used. HEK^TLR6^ cells were seeded in 24-well plates (50,000 cells per well) 1 day before transfection. Cells were transfected with pU6Rosa-CAG-Cas9, pTLR-donor template and one or two plasmids for the expression of CtIP or Cas9 fusion proteins (375 ng each) using Xtreme gene transfection reagent according to manufacturer’s recommendation. All samples were transfected in triplicate unless otherwise stated. FACS analysis for all the experiments was performed upon 72 h after transfection. For preparation of cells for FACS analysis the medium was aspirated from wells and each well was washed with PBS and treated with Trypsin for cell detachment. The cells were collected after adding media by centrifugation at 300 g for 4 min and finally resuspended in 500 μl PBS. The cells were kept on ice and FACS analysis was performed immediately. Single cells were gated for BFP positive populations and the frequency of Venus (HDR) and RFP (NHEJ) positive cells was determined using a BD LSR Fortessa flow cytometer (BD Biosciences). The results from replicate wells of each sample were used to calculate the mean value and standard deviation (SD). Mean values and SD were used to calculate *p*-values (*T*-test) using the GraphPad Prism 8 software (GraphPad Software Inc., San Diego, CA, United States).

### Synthetic Oligodeoxynucleotides

Synthetic oligonucleotides used in this study are shown in Table 1.

## Author Contributions

N-TT, SB, KR, and RK conceived and designed the project. N-TT, JR, and SB acquired the data. N-TT, SB, and RK analyzed and interpreted the data. VTC, XL, and JR provided materials. N-TT, SB, KR, and RK wrote the manuscript.

## Conflict of Interest Statement

The authors declare that the research was conducted in the absence of any commercial or financial relationships that could be construed as a potential conflict of interest.

## Footnotes

1 https://crisprgold.mdc-berlin.de/

## References

[B1] Barrangou R. Doudna J. A. (2016). Applications of CRISPR technologies in research and beyond. *Nat. Biotechnol.* 34 933–941. 10.1038/nbt.3659 27606440

[B2] Bunting S. F. Callén E. Wong N. Chen H.-T. Polato F. Gunn A. (2010). 53BP1 inhibits homologous recombination in *Brca1*-deficient cells by blocking resection of DNA breaks. *Cell* 141 243–254. 10.1016/j.cell.2010.03.012 20362325 PMC2857570

[B3] Canny M. D. Moatti N. Wan L. C. K. Fradet-Turcotte A. Krasner D. Mateos-Gomez P. A. (2018). Inhibition of 53BP1 favors homology-dependent DNA repair and increases CRISPR-Cas9 genome-editing efficiency. *Nat. Biotechnol.* 36 95–102. 10.1038/nbt.4021 29176614 PMC5762392

[B4] Charpentier M. Khedher A. H. Y. Menoret S. Brion A. Lamribet K. Dardillac E. (2018). CtIP fusion to Cas9 enhances transgene integration by homology-dependent repair. *Nat. Commun.* 9:1133. 10.1038/s41467-018-03475-7 29556040 PMC5859065

[B5] Chu V. T. Weber T. Wefers B. Wurst W. Sander S. Rajewsky K. (2015). Increasing the efficiency of homology-directed repair for CRISPR-Cas9-induced precise gene editing in mammalian cells. *Nat. Biotechnol.* 33 543–548. 10.1038/nbt.3198 25803306

[B6] Daley J. M. Sung P. (2014). 53BP1, BRCA1, and the choice between recombination and end joining at DNA double-strand breaks. *Mol. Cell. Biol.* 34 1380–1388. 10.1128/MCB.01639-13 24469398 PMC3993578

[B7] Escribano-Díaz C. Orthwein A. Fradet-Turcotte A. Xing M. Young J. T. F. Tkáč J. (2013). A cell cycle-dependent regulatory circuit composed of 53BP1-RIF1 and BRCA1-CtIP controls DNA repair pathway choice. *Mol. Cell* 49 872–883. 10.1016/j.molcel.2013.01.001 23333306

[B8] Fradet-Turcotte A. Canny M. D. Escribano-Díaz C. Orthwein A. Leung C. C. Y. Huang H. (2013). 53BP1 is a reader of the DNA-damage-induced H2A Lys 15 ubiquitin mark. *Nature* 499 50–54. 10.1038/nature12318 23760478 PMC3955401

[B9] Gupta A. Hunt C. R. Chakraborty S. Pandita R. K. Yordy J. Ramnarain D. B. (2014). Role of 53BP1 in the regulation of DNA double-strand break repair pathway choice. *Radiat. Res.* 181 1–8. 10.1667/RR13572.1 24320053 PMC4133096

[B10] Gutschner T. Haemmerle M. Genovese G. Draetta G. F. Chin L. (2016). Post-translational regulation of Cas9 during G1 enhances homology-directed repair. *Cell Rep.* 14 1555–1566. 10.1016/j.celrep.2016.01.019 26854237

[B11] Howden S. E. McColl B. Glaser A. Vadolas J. Petrou S. Little M. H. (2016). A Cas9 variant for efficient generation of indel-free knockin or gene-corrected human pluripotent stem cells. *Stem Cell Rep.* 7 508–517. 10.1016/j.stemcr.2016.07.001 27499201 PMC5031952

[B12] Huertas P. Jackson S. P. (2009). Human CtIP mediates cell cycle control of DNA end resection and double strand break repair. *J. Biol. Chem.* 284 9558–9565. 10.1074/jbc.M808906200 19202191 PMC2666608

[B13] Komor A. C. Badran A. H. Liu D. R. (2016). CRISPR-based technologies for the manipulation of eukaryotic genomes. *Cell* 168 20–36. 10.1016/j.cell.2016.10.044 27866654 PMC5235943

[B14] Konermann S. Brigham M. D. Trevino A. E. Joung J. Abudayyeh O. O. Barcena C. (2015). Genome-scale transcriptional activation by an engineered CRISPR-Cas9 complex. *Nature* 517 583–588. 10.1038/nature14136 25494202 PMC4420636

[B15] Lin S. Staahl B. T. Alla R. K. Doudna J. A. (2014). Enhanced homology-directed human genome engineering by controlled timing of CRISPR/Cas9 delivery. *eLife* 3:e04766. 10.7554/eLife.04766 25497837 PMC4383097

[B16] Liu J. Ehmsen K. T. Heyer W.-D. Morrical S. W. (2011). Presynaptic filament dynamics in homologous recombination and DNA repair. *Crit. Rev. Biochem. Mol. Biol.* 46 240–270. 10.3109/10409238.2011.576007 21599536 PMC4083101

[B17] Maruyama T. Dougan S. K. Truttmann M. C. Bilate A. M. Ingram J. R. Ploegh H. L. (2015). Increasing the efficiency of precise genome editing with CRISPR-Cas9 by inhibition of nonhomologous end joining. *Nat. Biotechnol.* 33 538–542. 10.1038/nbt.3190 25798939 PMC4618510

[B18] Mirman Z. Lottersberger F. Takai H. Kibe T. Gong Y. Takai K. (2018). 53BP1-RIF1-shieldin counteracts DSB resection through CST- and Polα-dependent fill-in. *Nature* 560 112–116. 10.1038/s41586-018-0324-7 30022158 PMC6072559

[B19] Orthwein A. Noordermeer S. M. Wilson M. D. Landry S. Enchev R. I. Sherker A. (2015). A mechanism for the suppression of homologous recombination in G1 cells. *Nature* 528 422–426. 10.1038/nature16142 26649820 PMC4880051

[B20] Paulsen B. S. Mandal P. K. Frock R. L. Boyraz B. Yadav R. Upadhyayula S. (2017). Ectopic expression of RAD52 and dn53BP1 improves homology-directed repair during CRISPR–Cas9 genome editing. *Nat. Biomed. Eng.* 1 878–888. 10.1038/s41551-017-0145-2 31015609 PMC6918705

[B21] Peabody D. S. Lim F. (1996). Complementation of RNA binding site mutations in MS2 coat protein heterodimers. *Nucleic Acids Res.* 24 2352–2359. 10.1093/nar/24.12.2352 8710507 PMC145953

[B22] Sartori A. A. Lukas C. Coates J. Mistrik M. Fu S. Bartek J. (2007). Human CtIP promotes DNA end resection. *Nature* 450 509–514. 10.1038/nature06337 17965729 PMC2409435

[B23] Savic N. Ringnalda F. C. Lindsay H. Berk C. Bargsten K. Li Y. (2018). Covalent linkage of the DNA repair template to the CRISPR-Cas9 nuclease enhances homology-directed repair. *eLife* 7:e33761. 10.7554/eLife.33761 29809142 PMC6023611

[B24] Shen M. W. Arbab M. Hsu J. Y. Worstell D. Culbertson S. J. Krabbe O. (2018). Predictable and precise template-free CRISPR editing of pathogenic variants. *Nature* 563 646–651. 10.1038/s41586-018-0686-x 30405244 PMC6517069

[B25] Symington L. S. (2016). Mechanism and regulation of DNA end resection in eukaryotes. *Crit. Rev. Biochem. Mol. Biol.* 51 195–212. 10.3109/10409238.2016.1172552 27098756 PMC4957645

[B26] Symington L. S. Gautier J. (2011). Double-strand break end resection and repair pathway choice. *Annu. Rev. Genet.* 45 247–271. 10.1146/annurev-genet-110410-132435 21910633

[B27] Tanenbaum M. E. Gilbert L. A. Qi L. S. Weissman J. S. Vale R. D. (2014). A protein-tagging system for signal amplification in gene expression and fluorescence imaging. *Cell* 159 635–646. 10.1016/j.cell.2014.09.039 25307933 PMC4252608

[B28] Uphoff C. C. Drexler H. G. (2002). Comparative PCR analysis for detection of mycoplasma infections in continuous cell lines. *In Vitro Cell. Dev. Biol. Anim.* 38 79–85. 11928999 10.1290/1071-2690(2002)038<0079:CPAFDO>2.0.CO;2

[B29] Yang D. Scavuzzo M. A. Chmielowiec J. Sharp R. Bajic A. Borowiak M. (2016). Enrichment of G2/M cell cycle phase in human pluripotent stem cells enhances HDR-mediated gene repair with customizable endonucleases. *Sci. Rep.* 6:21264. 10.1038/srep21264 26887909 PMC4757933

[B30] Zakharyevich K. Ma Y. Tang S. Hwang P. Y.-H. Boiteux S. Hunter N. (2010). Temporally and biochemically distinct activities of Exo1 during meiosis: double-strand break resection and resolution of double Holliday junctions. *Mol. Cell* 40 1001–1015. 10.1016/j.molcel.2010.11.032 21172664 PMC3061447

[B31] Zimmermann M. de Lange T. (2014). 53BP1: pro choice in DNA repair. *Trends Cell Biol.* 24 108–117. 10.1016/j.tcb.2013.09.003 24094932 PMC3946699

[B32] Zimmermann M. Lottersberger F. Buonomo S. B. Sfeir A. de Lange T. (2013). 53BP1 regulates DSB repair using Rif1 to control 5’ end resection. *Science* 339 700–704. 10.1126/science.1231573 23306437 PMC3664841

