# Corrigendum: Enhancement of Precise Gene Editing by the Association of Cas9 With Homologous Recombination Factors

**DOI:** 10.3389/fgene.2020.00326

**Published:** 2020-04-17

**Authors:** Ngoc-Tung Tran, Sanum Bashir, Xun Li, Jana Rossius, Van Trung Chu, Klaus Rajewsky, Ralf Kühn

**Affiliations:** ^1^Max-Delbrück-Centrum Für Molekulare Medizin, Berlin, Germany; ^2^Berlin Institute of Health, Berlin, Germany

**Keywords:** Cas9, gene editing, homologous recombination, CtIP, CRISPR

In the original article, there was a mistake made in Figure 2B as published. The construct at position 6 should show a fusion construct of Ctip-Mut with the N-terminal end of Cas9, instead of the C-terminal end. The corrected Figure 2B appears below.

**Figure 2B:**
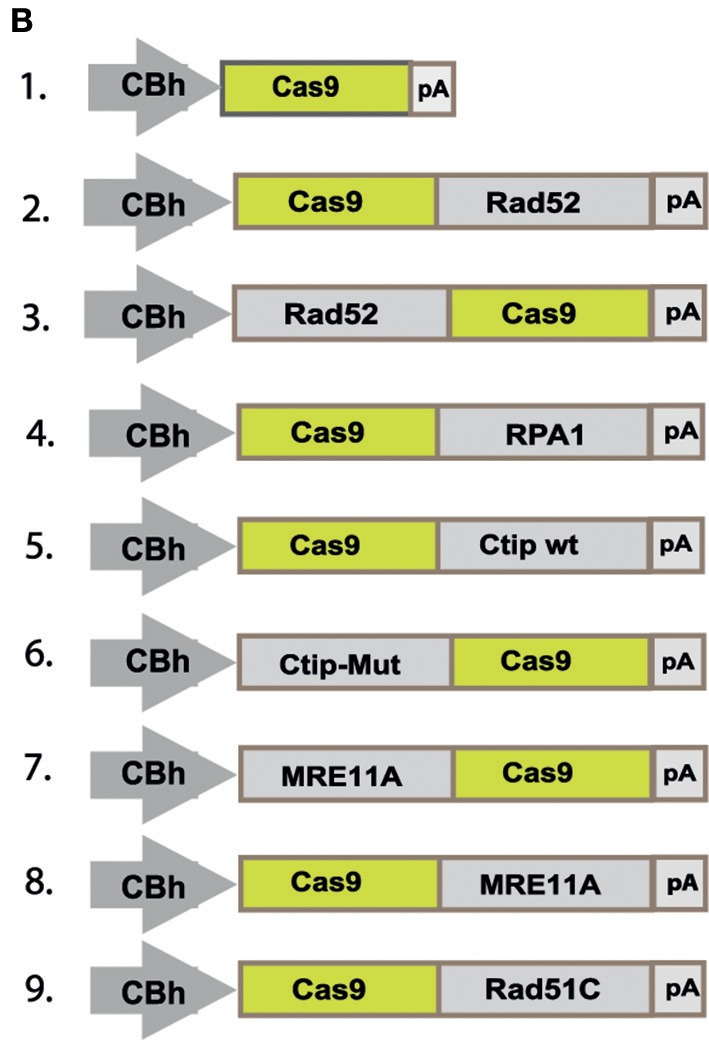


In Figure 4C “Cas9-Ctip mut” should be “Ctip-Mut-Cas9”. The corrected Figure 4C appears below.

**Figure 4C:**
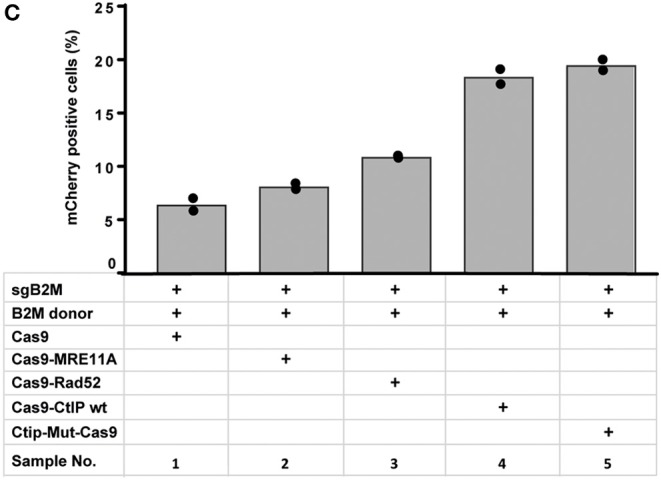


Finally, there is an error in the text describing Figure 2 refers to a C-terminal fusion of the CtIP (T847) mutant with Cas9. A correction has been made to the Results section, subsection DSB Repair Modification by Cas9 Fusion Proteins.

“To quantitatively determine CRISPR/Cas9-induced DSB repair by HDR or NHEJ, we used a traffic light reporter (TLR) construct, integrated into the AAVS1 locus of human HEK293 cells (HEK^TLR^) as previously described (Chu et al., 2015). Briefly, the reporter cassette includes a CAG promoter for expression of a non-functional coding region for Yellow fluorescent (Venus) protein, disrupted by the replacement of codons 117–152 with a 23 bp gRNA target sequence from the mouse *Rosa26* locus (sgRosa26), followed by a P2A peptide and the coding region for a red fluorescent (TagRFP) protein in a reading frame shifted by 2 bp (Figure 2A). If an intact Venus coding sequence is provided as a template and the repair of DSBs occurs via the HDR pathway the reporter cells are detected by the expression of Venus. CRISPR/Cas9-induced DSBs in the target region that are repaired via NHEJ and acquire Indels resulting into the shift of translation into the frame (+2) of P2A-RFP are detectable by the expression of RFP in reporter cells. Analysis by the inDelphi tool (Shen et al., 2018) for the repair of the *Rosa26* target site in HEK293 cells predicts a frequency of 16% of products in the +2 frame. Therefore, Venus positive reporter cells represent all HDR events but the number of RFP positive cells in a sample indicates only a fraction of NHEJ repair events. For the assessment of HDR modifiers we constructed N- or C-terminal fusions of Cas9 with the coding regions of human MRE11A, CtIP (wildtype or the phosphomimetic T847E mutant), RPA1, Rad51C, or Rad52 separated by a flexible linker of 16 residues (Figure 2B). To monitor the effects of Cas9 fusions on DSBs repair pathways, we co-transfected HEK^TLR^ cells with plasmids expressing either Cas9 or Cas9 fusions, a vector for expression of sgRosa26 and a Blasticidine resistance gene together with the donor plasmid (pTLR-repair) for repair of the defective Venus reporter gene (Figure 2C). The transfected cells were selected with Blasticidine for the enrichment of transfected cells and the frequency of Venus^+^ and RFP^+^ cells was analyzed 4 days later by flow cytometry (Supplementary Figure 1) in 4 independent samples. The results were used to calculate mean values and standard deviation. The ratio of Venus^+^ versus RFP^+^ cells is used as a relative index for DSB repair of the reporter by HDR or by NHEJ events resulting into the +2-reading frame. As shown in Figure 2D, upon expression of Cas9 we observed 0.95% of Venus^+^ and 7.55% of RFP^+^ cells in the ratio of 0.13 (sample 1). The expression of a N- or C-terminal fusion protein of Cas9 with Rad52 both lead to the increase of Venus^+^ and the decrease of RFP^+^ cells, shifting the Venus/RFP ratio to values of 0.35 and 0.42, respectively. The expression of a N- or C-terminal fusion protein of Cas9 with MRE11A lead to a higher increase of Venus^+^ cells but a lower decrease of RFP^+^ cells (samples 7 and 8), exhibiting Venus/RFP ratios of 0.35 and 0.37, respectively. The expression of a C-terminal fusion of Cas9 with CtIP or its N-terminal fusion with the CtIP (T847E) mutant (samples 5 and 6) increased the level of Venus^+^ cells up to 2.6%, showing Venus/RFP ratios of 0.42 and 0.53, respectively. In contrast to Rad52, MRE11A, and CtIP, the Cas9-RPA1 fusion protein (sample 4) lead to a smaller shift of the Venus/RFP ratio (0.22). As compared to the control (Cas9, sample 1) the increase of Venus^+^ cells and the decrease of RFP^+^ cells in samples 2–8 was significantly different (*p* < 0.05). Only the expression of the Cas9-Rad51C fusion (sample 9) resulted into levels of Venus^+^ cells and RFP^+^ cells that were not significantly different from the control (sample 1). These results show that the use of Cas9 fusions with multiple proteins of the HDR pathway, specifically CtIP, MRE11A and Rad52, can be used to stimulate DSB repair by HDR up to 2.7-fold.”

The authors apologize for these errors and state that these do not change the scientific conclusions of the article in any way. The original article has been updated.
