# Supplementary material for: Enhancement of Precise Gene Editing by the Association of Cas9 With Homologous Recombination Factors
Source: Front Genet. 2019 Apr 30;10:365. doi: 10.3389/fgene.2019.00365 (PMC6503098; doi:10.3389/fgene.2019.00365)

Enhancement of precise gene editing by the association of Cas9 with homologous recombination factors

Supplementary Figure 1: DSB repair modification by Cas9 fusion proteins

FACS plots representing the result from one sample of each transfection shown in Fig. 2, indicating the percentage of Venus and RFP positive cells within the population by the inserted numbers.

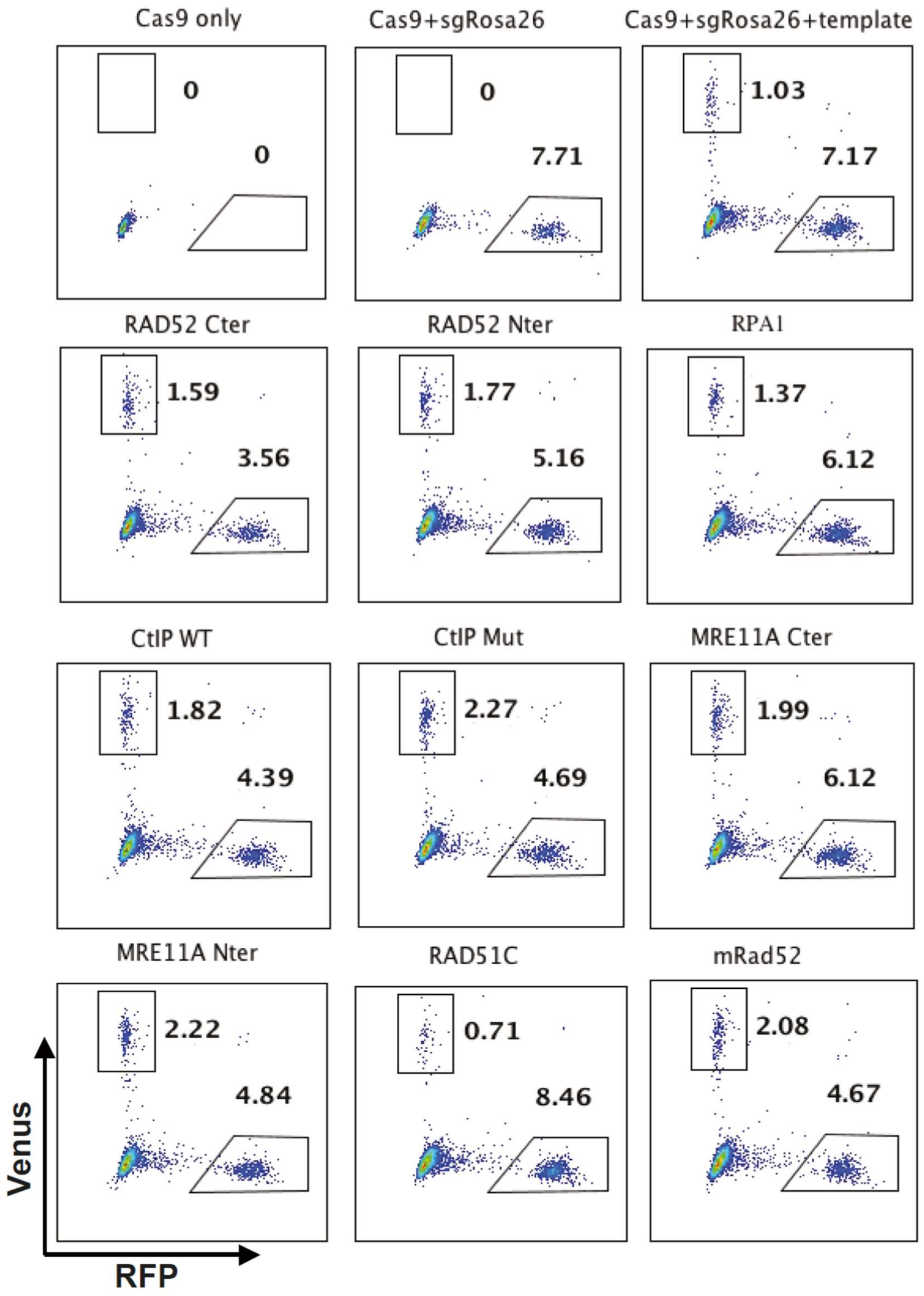

Supplementary Figure 2: Cas9 fusion proteins in 53BP1 knockout HEK^{TLR} cells

(A) sgRNAs targeting exon 1 or exon 2 of the 53BP1 gene were cloned into the pX330 expression vector and transfected into HEK cells. Genomic DNA was isolated, used for PCR amplification using exon specific primers and the PCR product was treated with T7 endonuclease (T7EI) and separated by gel electrophoresis. PCR product from untransfected cells (Ctrl) is not digested by T7EI, digestion products in the transfected samples (lane 1, 2) indicate the efficiency of sgRNAs to induce Indel formation by NHEJ repair. For the isolation of 53BP1 knockout cells both sgRNA vectors were cotransfected into HEK^{TLR} cells. Three days post-transfection, the transfected cells were sorted as single cells into 96-well plates, cultured and expanded for genotyping by PCR using primers T7_h53BP1_F and _R (Table 1). Deletion of the 53BP1 gene segment between the Cas9 target sites in the first and second exon was confirmed by PCR and sequencing of the PCR product. Clone 7 showed homozygous deletions and was used for further experiments. **(B)** FACS plots representing the result from one sample of each transfection shown in Fig. 3, indicating the percentage of Venus and RFP positive cells within the population by the inserted numbers.

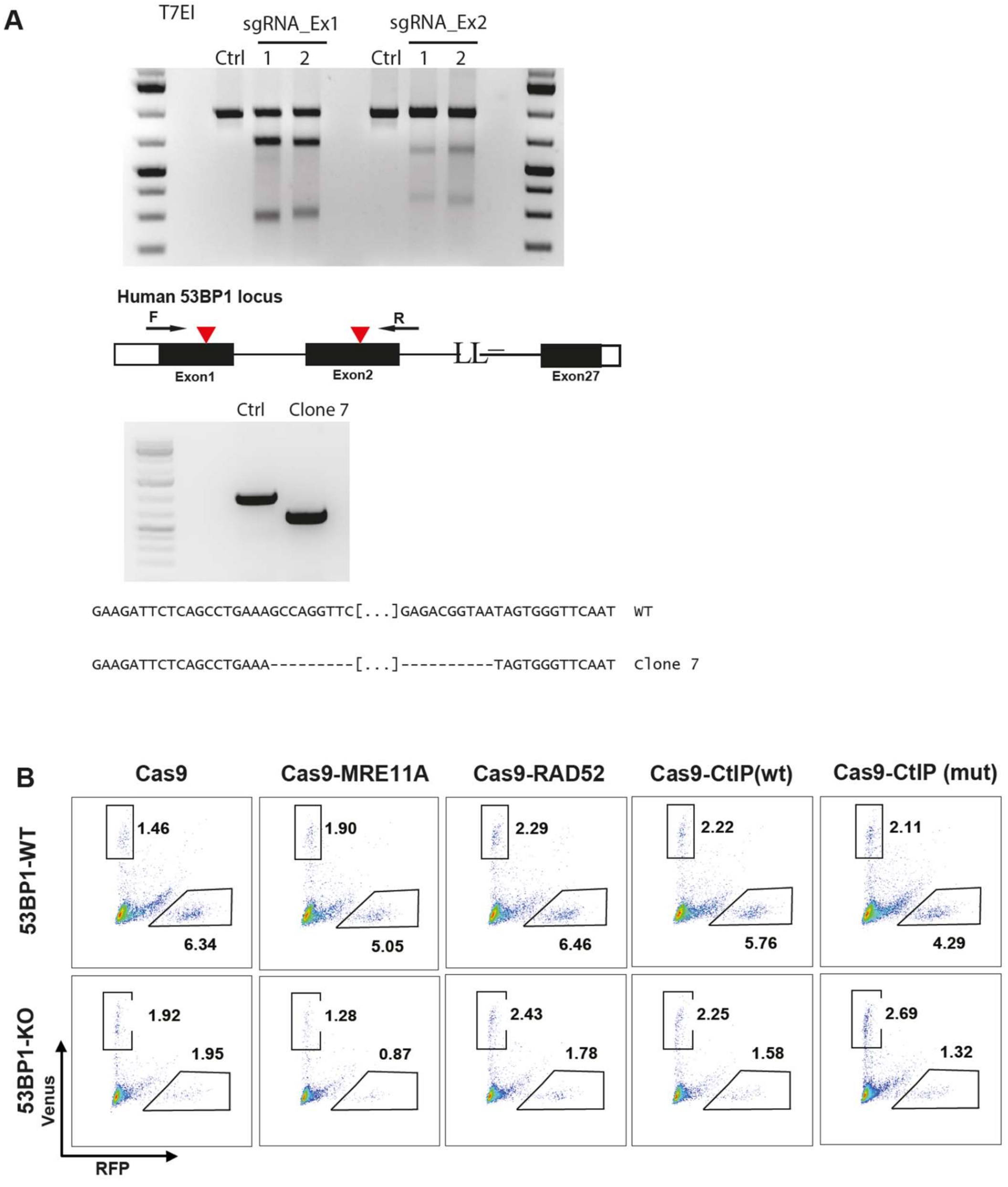

Supplementary Figure 3: Targeting of the beta-2 microglobulin gene

FACS plots representing the result from one sample of each transfection shown in Fig. 4, indicating the percentage of Cherry positive cells within the population by the inserted numbers.

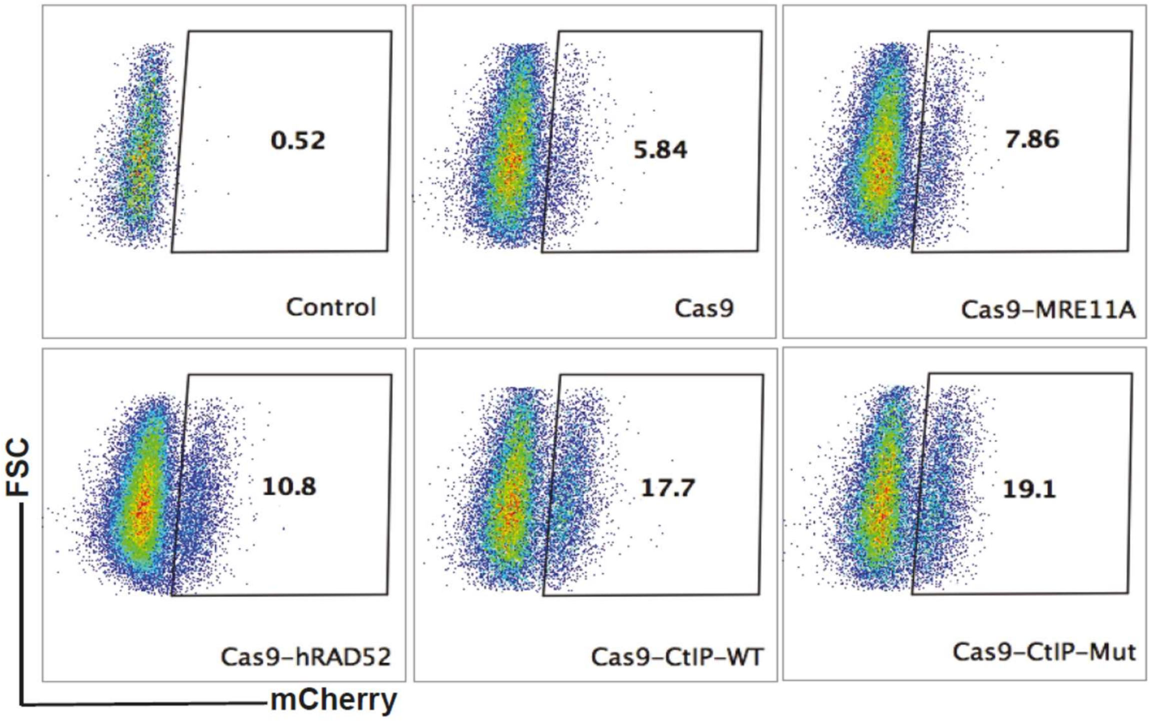

Supplementary Fig. 4: Structure of MS2 and SunTag fusion proteins binding to Cas9/gRNA complexes.

(A) Structure of guide RNA including two copies of a MS2 phage-derived RNA aptamer sequence added to the tetraloop and stem loop 2 of the gRNA scaffold that can be bound by the MS2 phage coat protein. **(B)** MS2 aptamer bound by a dimer of MS2 protein formed by association of two monomers. Each monomer is fused at its C-terminus to a protein of interest and a single apatmer motif is bound by two copies of the fusion protein. **(C)** MS2 aptamer bound by a single chain dimer of two genetically fused MS2 protein monomers that are connected via their N- and C-terminus ($MS2^{di}$). The C-terminus of the single chain dimer is fused to a protein of interest such that a single apatmer motif is bound by one copy of the fusion protein. **(D)** Structure of the Cas9 SunTag system: the C-terminus of Cas9 is fused with 10 copies of a yeast GCN4 protein-derived peptide sequence (residues 258-76), separated by Glycine/Serine linker. Each GCN4 peptide motif can be bound by the Sun-L ligand consisting of an anti-GCN4 single-chain antibody that is fused with a protein of interest; only two Sun-L molecules are depicted.

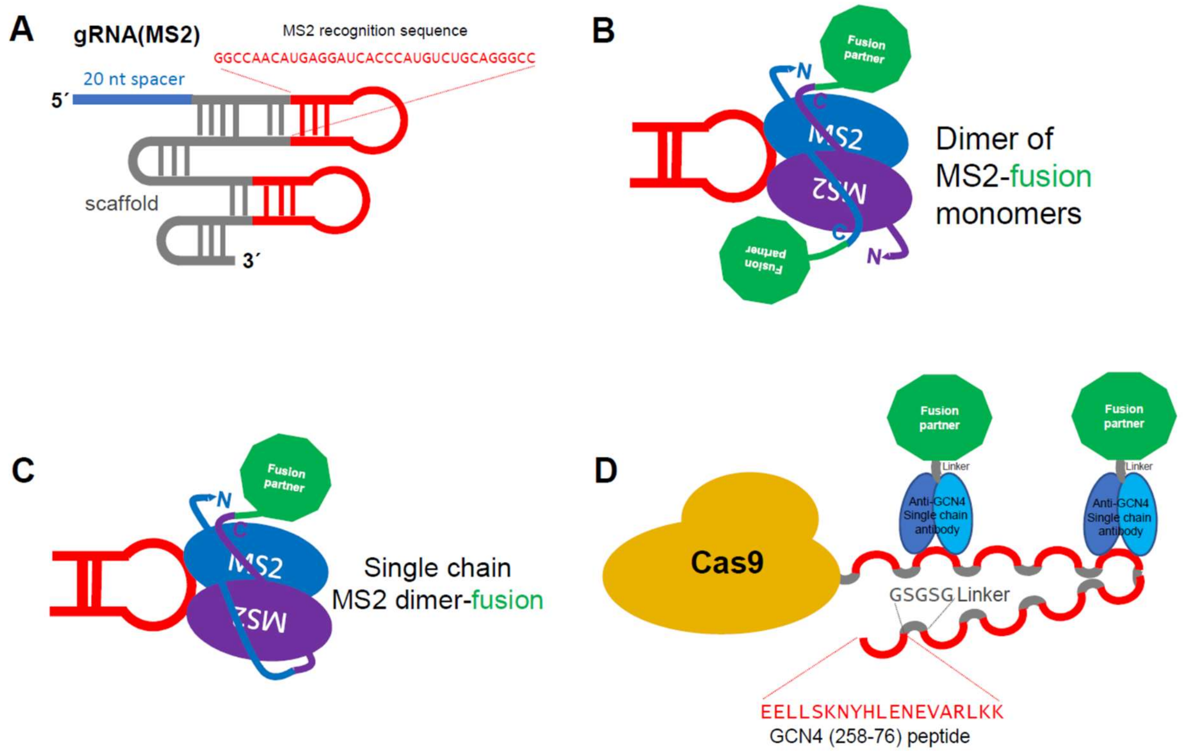

Supplement: Supplementary file 1 [file Data_Sheet_1.pdf]
